# Plasma proteomic markers predict risk for bowel resection in inflammatory bowel disease: a retrospective cohort study

**DOI:** 10.1097/JS9.0000000000004636

**Published:** 2026-01-12

**Authors:** Xinyi Xu, Chenyue Xu, Na Zeng, Qi Sun, Fang Xu, Jiuyue Ma, Xueping Huang, Minsi Zhou, Jing Sun, Shengtao Zhu, Peng Li, Shutian Zhang, Han Lv, Haiyun Shi

**Affiliations:** a Department of Gastroenterology, Beijing Friendship Hospital, Capital Medical University, Beijing, China; b State Key Laboratory of Digestive Health, Beijing, China; c National Clinical Research Center for Digestive Disease, Beijing, China; d Beijing Key Laboratory of Early Gastrointestinal Cancer Medicine and Medical Devices, Beijing, China; e School of Public Health, Peking University, Beijing, China; f Department of Imaging, Beijing Friendship Hospital, Capital Medical University, Beijing, China

**Keywords:** bowel resection, inflammatory bowel disease, plasma proteomics

## Abstract

**Background:**

Bowel resection is required in up to 27% of inflammatory bowel disease (IBD) patients due to severe complications. Identifying reliable predictors for future surgery is crucial for risk stratification and management.

**Materials and Methods:**

We retrospectively analyzed UK Biobank data of IBD patients with plasma proteomics measured using the Olink platform. A Random Survival Forest (RSF) model was used to evaluate the predictive performance of inflammatory proteins for surgical risk. The concordance index (C-index) compared Olink-based and conventional clinical models. A predicted risk score quantified individual surgery risk, and Kaplan-Meier analysis assessed differences in surgery-free survival between high- and low-risk groups. Cox proportional hazards models verified the independent predictive value of proteomics.

**Results:**

Among 915 patients with IBD, 81 cases underwent bowel resection during follow-up. The RSF model using Olink proteins achieved a superior C-index compared to conventional markers (0.784 vs 0.543, *P* < 0.01). Key predictors included IL15, PAPPA, and PIK3AP1. Patients with a higher predicted risk score had a significantly higher surgery risk (*P* < 0.0001). The predicted risk score remained an independent predictor for surgery risk [adjusted HR: 0.83, 95% confidence interval (CI): 0.81–0.86]. The net reclassification analysis demonstrated improved patient-level stratification by proteomic features beyond standard clinical predictors (net reclassification improvement: 0.096, 95% CI: 0.008–0.257, *P* = 0.027).

**Conclusion:**

Plasma proteomic markers demonstrated superior predictive performance over traditional markers in assessing future bowel resection risk in IBD. Incorporating proteomics into clinical practice could enhance risk stratification and inform early interventions to reduce surgical burdens.

## Introduction

Inflammatory bowel disease (IBD), including ulcerative colitis (UC) and Crohn’s disease (CD), is a chronic, relapsing inflammatory disorder of the gastrointestinal tract. The global prevalence of IBD continues to rise, posing a growing health care burden and reflecting significant challenges in disease management^[1,2]^. A substantial proportion of IBD patients experience severe complications during their disease course, including intestinal strictures, obstruction, perforation, fistulas, bleeding, and malignancy, often necessitating surgical intervention and resulting in further complications, including short bowel syndrome^[3,4]^.

Despite advancements in pharmacological therapies, surgery rates in IBD remain considerable. Recent data show that approximately 6%–14% of CD patients undergo surgery within 1 year and 12%–27% within 5 years^[5]^. For UC, surgery rates are 1%–5% at 1 year and 3%–8% at 5 years. While early biological therapy has shown potential to reduce surgery rates in CD [OR: 0.63; 95% confidence interval (CI): 0.48–0.84]^[6]^, its impact in UC is unclear. Early biological use in UC has been associated with higher colectomy rates (OR: 2.86; 95% CI: 1.30–6.30), possibly due to confounding by indication, as patients with severe disease are more likely to receive early biological therapy.

Several clinical predictors of surgery have been identified. In CD, stricturing or penetrating disease behavior, younger age at diagnosis, and smoking status are strongly associated with an elevated need for surgery^[7]^. These factors often result in recurrent surgeries and complications, presenting further management challenges. In UC, factors such as proximal disease extension, primary sclerosing cholangitis, and steroid-dependent increase colectomy needs^[7]^. In addition to clinical factor risk stratification, optimal clinical care – including early involvement of nutrition specialists – may influence long-term outcomes in IBD patients, as nutritional status is closely linked to disease course^[8]^.

However, accurately identifying patients at high risk of surgery remains a key clinical challenge^[9]^. Traditional inflammatory markers, such as C-reactive protein (CRP) and fecal calprotectin, lack the specificity required to capture the dynamic complexity of IBD-related complications^[10]^. Advances in proteomics, particularly the proximity extension assay (PEA) technology, provide a promising avenue for identifying novel biomarkers with improved clinical utility^[11]^.

In this study, we leveraged plasma proteomics data generated by the Olink platform within the UK Biobank to assess its utility in predicting the risk of future bowel resection among IBD patients. This cohort study has been reported in line with the STROCSS guidelines^[12]^.

## Materials and methods

### Study population

This study was a retrospective observational analysis based on the UK Biobank, a prospective population-based cohort comprising over 500 000 participants aged 40–69 years at recruitment. Participants with a confirmed diagnosis of IBD, including CD or UC, were identified based on linked medical records using the ICD10 codes K50 and K51.

Proteomic profiling using the Olink Explore platform was conducted in approximately 54 000 participants (~10% of the full UK Biobank cohort) as part of the UK Biobank Pharma Proteomics Project (UKB-PPP). The UKB-PPP cohort included a randomly selected subset of 46 595 participants at baseline, 6376 individuals selected by consortium members based on specific disease phenotypes, and 1268 participants who took part in the COVID-19 repeat-imaging study^[13]^. According to the UKB-PPP quality control report^[13]^, the UKB-PPP cohort was demographically and clinically representative of the broader UK Biobank population. The prevalence of UC and CD in the proteomic subset was nearly identical to that of the full UK Biobank cohort (UC 1.32% vs 1.25%, *P* = 0.18; CD 0.72 % vs 0.65 %, *P* = 0.05), indicating no substantial enrichment bias.

Inclusion criteria for the present analysis were (1) a confirmed diagnosis of IBD, and (2) availability of baseline plasma proteomics data. Exclusion criteria included (1) a history of bowel resection (e.g., ileal resection or colectomy) prior to the baseline assessment. A flowchart outlining the derivation of the analytic cohort is provided in Supplemental Digital Content Figure S1, available at: http://links.lww.com/JS9/G536.

### Data collection

Clinical data, including demographic variables (age, gender, and smoking status), laboratory parameters [CRP, albumin, platelet count, hemoglobin (Hb), and white blood cell (WBC) count], and medication history, were collected from baseline visits. Plasma protein levels were quantified using the Olink platform, which employs PEA technology for high-throughput and precise measurement of proteins. From the 2923 protein assays available, we selectively used the inflammation panel, which measures 92 inflammation-related proteins (Supplemental Digital Content Table S1, available at: http://links.lww.com/JS9/G537) listed in the OLINK EXPLORE 1536 platform (Uppsala, Sweden), as these proteins were biologically relevant to IBD and consistently measured across all included participants. Normalized Protein Expression (NPX, log2 scale) values were used for statistical analysis.

**HIGHLIGHTS**

- Plasma proteomics significantly outperformed conventional clinical markers in predicting future bowel resection among inflammatory bowel disease (IBD) patients.
- Key inflammatory proteins, including IL15, PAPPA, and PIK3AP1, were identified as strong predictors of surgical risk.
- A proteomics-based risk score independently stratified patients by surgery risk, supporting its potential for personalized IBD management.

### Statistical analysis

We first assessed missingness across baseline variables (Supplemental Digital Content Table S2, available at: http://links.lww.com/JS9/G538). Little’s MCAR test (MissMech package in R) indicated data were either Missing Completely at Random (MCAR) or at Random (MAR). Missing values of continuous clinical data were imputed using the k-nearest neighbors (kNN) function from the VIM R package, while categorical variables were imputed using mode imputation^[14]^. Missing values of proteomic data were imputed using Bayesian ridge regression using scikit-learn Python package^[15]^. Baseline characteristics of the study population were compared using R version 4.4.0. Continuous variables were analyzed using Student *t*-tests or Wilcoxon rank-sum tests, while categorical variables were compared using χ^2^ test or Fisher’s exact test. Normally distributed data with homogeneous variance were presented as mean ± SD, while non-normally distributed data were expressed as medians with interquartile range.

To evaluate potential IBD subtype-specific variations, we performed principal coordinates analysis (PCoA) incorporating all 388 inflammatory proteins from the Olink panel. The analysis revealed no distinct clustering between UC and CD cases (Supplemental Digital Content Figure S2, available at: http://links.lww.com/JS9/G536), indicating substantial overlap in their inflammatory protein profiles^[16]^. Based on these findings, we developed a unified predictive model to identify common molecular signatures associated with surgical risk across both IBD subtypes. Random Survival Forest (RSF) model, implemented in Python using the scikit-survival package with fivefold cross-validation, was applied to predict the risk of bowel resection. Three predictive models were constructed: (1) Model 1: Olink-based proteins. (2) Model 2: Conventional clinical markers (CRP, albumin, platelet count, Hb, and WBC count). (3) Model 3: Combination of the Olink-based top 30 proteins that reached maximum predictive accuracy and conventional clinical markers. Model performance was measured using the concordance index (C-index) to evaluate the predictive accuracy for future bowel resection. Calibration was assessed by plotting the observed event rates against the predicted risks (1 – survival probability) at the median follow-up time (*t* = 6.9 years). Predicted probabilities were grouped by deciles to generate the calibration curve. To assess clinical utility, we conducted a net reclassification improvement (NRI) and integrated discrimination improvement (IDI) analysis at the 5-year time point using the survIDINRI R package.

Based on Model 1, a predicted risk score was calculated for each patient using the model-derived survival function. At follow-up time *t*, predicted survival probabilities were transformed into continuous risk scores, where higher values indicate a higher predicted risk of bowel resection. This score reflects the cumulative risk estimated by the trained RSF model and provides a quantitative measure of individualized surgical risk. For clinical interpretability, Kaplan-Meier survival curves were used to stratify patients into high- and low-predicted risk score groups using the median risk score as the cutoff, with statistical significance determined by the log-rank test. To evaluate the robustness of the risk stratification, sensitivity analyses were conducted by stratifying patients based on key clinical variables, including age (≤60 vs >60 years), disease duration (≤9.6 vs >9.6 years), and medication history [without vs with steroids or immunomodulators (IMMs)]. Within each subgroup, patients were further clustered by proteomic risk clusters, and Kaplan-Meier curves were generated to assess differences in bowel resection risk using the log-rank test. Cox proportional hazards (CPH) models, adjusted for variables such as age, IBD subtypes, smoking status, and demographic factors, were used to validate the independent predictive value of proteomics. Proportional hazards assumptions were tested using Schoenfeld residuals via cox.zph() (survival package, R). Adequate statistical power (90.5%) was confirmed using powerEpiCont R package based on cohort size, event rate, and hazard ratio (HR).

All analyses were conducted using R (version 4.4) and Python (version 3.11.8), with a threshold of *P* < 0.05 considered statistically significant. Key R packages included: *VIM* (v6.2.2) for kNN imputation, *MissMech* (v1.0.2) for Little’s MCAR test, *survival* (v3.5-7) for Cox modeling and proportional hazards testing via *cox.zph(), survIDINRI* (v1.1) for NRI and IDI calculations, and *powerSurvEpi* (v0.1.3) for power analysis. Python-based modeling employed *scikit-survival* (v0.22.0) for the RSF and generated calibration plots and *scikit-learn* (v1.4.2) for Bayesian ridge regression imputation.

## Results

### Baseline characteristics

From the initial cohort of 502 357 participants, 9156 individuals with a diagnosis of IBD were identified. After excluding 8145 patients without baseline proteomics data and 96 patients with prior bowel resection, 915 patients were included in the final analysis, of whom 81 underwent bowel resection during the follow-up period (Supplemental Digital Content Figure S1, available at: http://links.lww.com/JS9/G536). CD patients underwent higher surgery rates compared to UC (11.86% vs 7.42%, *P* = 0.037; Table 1). Patients who were current smokers had a significantly higher risk of requiring surgery compared to non-smokers or former smokers (18.52% vs 9.47%, *P* = 0.029; Table 2). The predicted risk score, representing the probability of remaining surgery-free during the follow-up period, was significantly higher in the surgery group compared to the non-surgery group [7.58 (5.59, 9.08) vs 2.70 (1.72, 4.06), *P* < 0.001). No significant differences were found in age, sex, BMI, IBD duration, alcohol consumption, or IMM use between the groups. Among conventional clinical markers, only CRP was found to be elevated in the surgery group at baseline. The median time between serum sample collection and subsequent bowel resection was 6.9 (IQR 2.7–9.5) years. A comparison of baseline characteristics between IBD patients with and without available Olink data is provided in Supplemental Digital Content Table S3, available at: http://links.lww.com/JS9/G539. Apart from a slightly higher mean age in the proteomic subset, no substantial differences were observed.

**Table 1:** Surgery condition stratified by IBD subtype.

	CD	UC	*P*
	(*n* = 295)	(*n* = 620)	
Surgery condition			
Without bowel resection	260 (88.14%)	574 (92.58%)	0.037*
With bowel resection	35 (11.86%)	46 (7.42%)	

Values are presented as number (%).

This table describes the surgery condition of IBD patients. Significant group differences were determined by χ^2^ test for categorical variables.

* *P* < 0.05.

**Table 2:** Baseline characteristics of enrolled patients.

	Without bowel resection	With bowel resection	*P*
	(*n* = 834)	(*n* = 81)	
Sex ratio (M:F)			
Female	435 (52.16%)	40 (49.38%)	0.718
Male	399 (47.84%)	41 (50.62%)	
Age (year)	60.00 (52.25, 64.00)	61.00 (54.00, 64.00)	0.421
Ethnicity			
Non-White	29 (3.48%)	2 (2.47%)	0.875
White	805 (96.52%)	79 (97.53%)	
BMI^a^	27.32 (4.60)	27.68 (4.31)	0.497
IBD duration (year)	9.90 (5.20, 19.28)	8.00 (4.00, 13.20)	0.063
Current smoker	79 (9.47%)	15 (18.52%)	0.018*
Alcohol drinker status			
Current	750 (89.93%)	70 (86.42%)	0.597
Never	41 (4.92%)	5 (6.17%)	
Previous	43 (5.16%)	6 (7.41%)	
5-ASA^b^ use	208 (24.94%)	28 (34.57%)	0.079
Steroid use	65 (7.79%)	6 (7.41%)	1
IMM^c^ use	65 (7.79%)	11 (13.58%)	0.112
CRP^d^	1.77 (0.92, 3.88)	2.36 (1.48, 4.04)	0.036*
ALB^e^	38.67 (37.02, 40.12)	38.48 (37.02, 40.00)	0.783
Hb^f^	14.10 (13.23, 15.10)	14.02 (13.09, 14.90)	0.415
WBC^g^	6.88 (6.00, 8.10)	6.63 (6.07, 7.90)	0.918
Platelet count	251.10 (213.10, 296.88)	254.30 (227.00, 294.40)	0.504
Predicted risk score	2.70 (1.72, 4.06)	7.58 (5.59, 9.08)	<0.001*

This table describes the baseline characteristics of IBD patients. Significant group differences were determined by using Student *t*-tests or Wilcoxon rank-sum tests (non-normal distributions) for continuous variables and χ^2^ or Fisher’s exact test (non-normal distributions) for categorical variables. Values are presented as median (IQR), or number (%).

* *P* < 0.05.

^a^ Body mass index,

^b^ 5-Aminosalicylic acid.

^c^ Immune modulators.

^d^ C-reactive protein.

^e^ Albumin.

^f^ Hemoglobin.

^g^ White cell count,

### Predictive models for surgery risk

**Figure 1.**
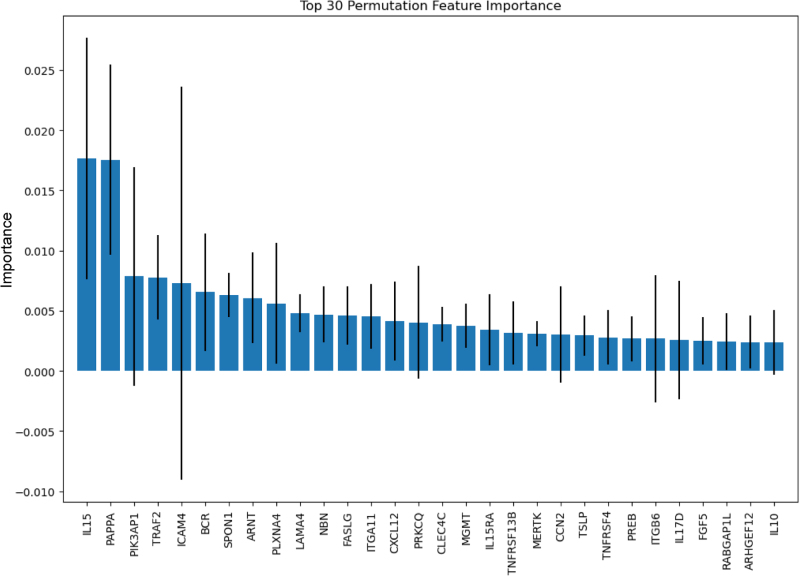
Top 30 proteins associated with future surgery occurrence. Permutation importance was calculated to assess the contribution of each plasma protein to the prediction of bowel resection in IBD patients. The top 30 proteins are ranked in descending order of mean importance, with error bars representing the standard deviation across multiple permutations.

The RSF model using 30 top-importance inflammation-related proteins demonstrated the highest predictive accuracy with a C-index of 0.784, significantly outperforming the model based on the panel of conventional clinical markers (C-index: 0.543, *P* < 0.01). The combination of Olink proteomics with clinical markers showed slightly lower predictive power (C-index: 0.737). The RSF model identified IL15, PAPPA, and PIK3AP1 as the top predictors for bowel resection (Fig. 1). None of IL15, PAPPA, or PIK3AP1 was correlated with age (Supplemental Digital Content Figure S3, available at: http://links.lww.com/JS9/G536). The RSF model demonstrated good calibration, with predicted risks closely matching observed outcomes at the median follow-up time (*t* = 6.9 years; Supplemental Digital Content Figure S4, available at: http://links.lww.com/JS9/G536).

To evaluate the clinical utility of the proteomics-based model, we performed an NRI analysis comparing the proteomics model to a conventional clinical model. At the 5-year time point, the presence of proteomic features significantly improved risk classification, with an IDI of 0.404 (95% CI: 0.135–0.601, *P* < 0.001) and an NRI of 0.096 (95% CI: 0.008–0.257, *P* = 0.027). These results demonstrate that the proteomic model is superior in risk stratification compared to traditional clinical predictors.

### Survival analysis and multivariate analysis

**Figure 2.**
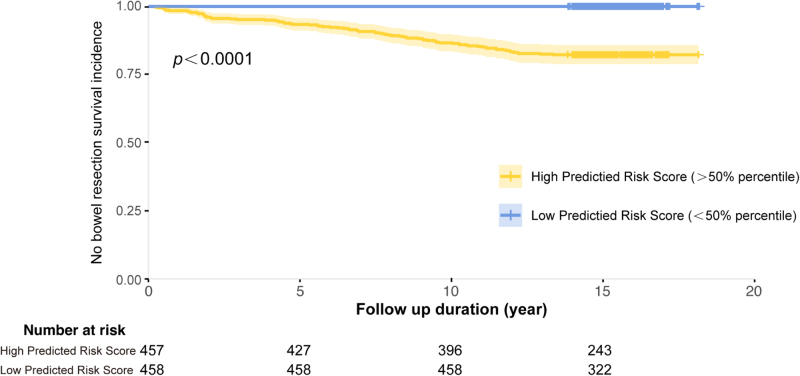
Kaplan-Meier survival curve comparing bowel resection-free survival between high predicted risk score (>50th percentile) and low predicted risk score (<50th percentile) groups.

Kaplan-Meier analysis showed that patients with higher predicted risk scores (>50th percentile) had a significantly higher surgery risk compared to those with lower scores (<50th percentile, *P* < 0.0001; Fig. 2). Sensitivity analyses stratified by key clinical variables demonstrated consistent and robust findings. The prognostic separation between proteomic-defined risk clusters remained statistically significant within both age subgroups (≤60 vs >60 years; Supplemental Digital Content Figure S5A, available at: http://links.lww.com/JS9/G536). Similarly, risk stratification remained significant when patients were grouped by disease duration (≤9.6 vs >9.6 years) and by medication history at baseline (without vs with steroids or IMMs; Supplemental Digital Content Figures S5B-C, available at: http://links.lww.com/JS9/G536).

**Figure 3.**
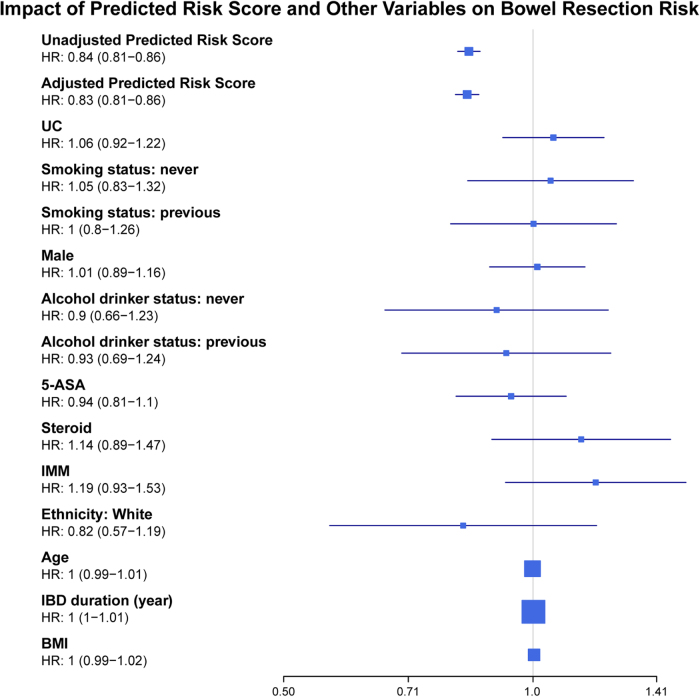
Forest plot showing the impact of predicted risk score and other clinical variables on bowel resection risk, as determined by the Cox proportional hazards model.

Multivariate analysis using a CPHs model confirmed that the predicted risk score was an independent predictor of surgery risk (adjusted HR: 0.83, 95% CI: 0.81–0.86; Fig. 3). The reported HR represents the risk associated with each one-unit increase in the predicted risk score. We also confirmed that the proportional hazards assumption was not violated, based on the results of the Schoenfeld residuals test (global and individual *P* > 0.05).

## Discussion

This study demonstrates the clinical potential of plasma proteomics for predicting surgical risk in IBD patients. Using the UK Biobank cohort, we showed that inflammatory proteomic markers significantly outperformed conventional clinical parameters in predicting surgical outcomes, with the RSF model achieving a C-index of 0.784. Among the 30 top-ranked inflammatory proteins, IL15, PAPPA, and PIK3AP1 emerged as key predictors, providing new insights into IBD pathogenesis and surgical risk mechanisms. These findings establish a framework for implementing molecular biomarkers in clinical practice and represent an important advance toward precision medicine in IBD management. By identifying specific inflammatory proteins associated with surgical risk, our work addresses a critical unmet need for predictive biomarkers in IBD while offering new perspectives on disease monitoring and therapeutic targeting.

Current biomarkers, such as CRP, erythrocyte sedimentation rate (ESR), and FCP, are widely used but limited in surgical prognostication. CRP, although a commonly used marker of inflammation in clinical settings^[17,18]^, often correlates poorly with endoscopic disease activity^[19]^. On day 3 of corticosteroid treatment in acute severe UC, a CRP threshold of >45 mg/L, combined with stool frequency between 3 and 8/day, predicted colectomy with a positive predictive value of 85%^[20]^. ESR is influenced by non-inflammatory factors like anemia and age, reducing its specificity^[21]^. While FCP has shown superiority in predicting mucosal inflammation, it exhibits variability in isolated ileal CD and is influenced by other gastrointestinal conditions, further limiting its predictive utility^[22–24]^. When assessing the risk of IBD-related surgery, an FCP threshold of 244 µg/g yielded a sensitivity of 65% and specificity of 65%, reaching an area under the curve (AUC) of 0.59^[25]^. Another research suggested that an FCP threshold of 1922.5 µg/g yielded a sensitivity of 24%, specificity of 97.4%, and a likelihood ratio of 9.23 during initial admission, while 87% of patients with FCP above this threshold required colectomy during a median follow-up of 1.1 years. These limitations reinforce the need for more robust biomarkers for disease activity and progression^[10]^.

Integrating proteomics into clinical workflows offers unique opportunities for patient care. Proteomic signatures have demonstrated promise in preclinical studies, with evidence suggesting their ability to predict IBD onset long before clinical diagnosis^[26]^. In CD, proteomics achieved an AUC of 0.85 in differentiating future patients from controls, while the predictive capacity for UC, though lower, remains significant (AUC 0.77). Beyond disease onset, serum proteomic panels, such as ITGAV, EpCAM, IL18, SLAMF7, and IL8, have shown utility in predicting treatment escalation, with an HR of 3.90 (95% CI: 2.43–6.26)^[27]^. In our study, the median interval between serum sample collection and subsequent bowel resection was 6.9 (IQR 2.7–9.5) years, well demonstrating the long-term predictive capacity of our model. It could identify early molecular risk signatures that precede clinical deterioration by several years. Prior research has shown that baseline plasma proteomes can predict IBD development and future disease escalation years in advance^[26–28]^, supporting the value of early molecular risk stratification.

Our identification of IL15, PAPPA, and PIK3AP1 as key predictors of surgical risk in IBD patients not only highlights the potential of plasma proteomics in risk stratification but also offers insights into the molecular mechanisms underlying disease progression and complications. IL15, a pro-inflammatory cytokine, is integral to the activation, proliferation, and maintenance of T cells and natural killer (NK) cells. IL15 activity was elevated in rectal mucosa in active IBD and even in inactive UC, and its elevation was also linked to relapses in IBD^[29,30]^. In mice colitis induced by dextran sodium sulfate, IL15 plays a crucial role in promoting intestinal inflammation by supporting the survival and activation of CD8^+^ T cells and NK cells, leading to the production of pro-inflammatory cytokines^[31]^. PAPPA, a metalloproteinase that enhances local IGF bioavailability, facilitates tissue repair by promoting cellular proliferation, differentiation, and survival^[32]^. A study indicates that PAPPA expression is elevated in the inflamed intestinal tissues of IBD patients, contributing to increased local IGF-I activity, which may play a role in tissue repair processes^[33]^. PIK3AP1, also known as BCAP (B cell adaptor for PI3K), is a signaling molecule that activates the phosphoinositide-3-kinase (PI3K) pathway, which regulates cell survival, proliferation, and metabolism^[34]^. Mutations in the PIK3AP1 gene were identified in 10% of UC cases, 20% of colorectal cancer cases, and 30% of colitis-associated colorectal cancer cases^[35]^. PIK3AP1 has been proven to be related to glucocorticoid-resistant patients with UC, and our study also confirms its role in predicting future surgery risk^[36]^. These findings suggest that proteomic IL15, PAPPA, and PIK3AP1 can complement existing tools, bridging the gap between clinical and molecular insights into IBD progression and treatment response. For instance, the protein IL17D was previously linked to treatment escalation^[26]^. Our study confirms this finding, further supporting the role of proteomics in refining risk stratification. Although CD and UC represent clinically distinct entities, we conducted PCoA using all 388 inflammatory proteins from the Olink panel to evaluate whether CD and UC exhibit distinct circulating proteomic signatures. The results demonstrated substantial overlap between CD and UC, with no distinct clustering differentiating the two subtypes. These findings were consistent with previous studies, suggesting that IBD exists on a molecular continuum rather than as two fully dichotomous inflammatory states^[16,37]^.

These proteomic signatures could guide risk-stratified monitoring, thereby enabling more frequent surveillance of high-risk patients. They could also support earlier or optimized therapeutic escalation, including the timely initiation of biologics or small molecular drugs for those with high predicted surgical risk. In addition, these markers could help refine clinical trials, enabling molecular stratification in patient recruitment for targeted therapy research.

While our results are encouraging, several limitations warrant consideration. First, although missingness was confirmed as MCAR/MAR and imputed appropriately, potential residual bias or distortion of true associations cannot be excluded. Among all analyzed variables, albumin exhibited the highest proportion of missing data. However, since albumin was not incorporated into our primary predictive model (Model 1), which was based exclusively on proteomic biomarkers for surgical risk prediction, this missingness did not affect the primary outcome. Second, since detailed clinical information was unavailable in the UK Biobank dataset, potential unmeasured confounders, such as baseline disease severity (e.g., clinical scores or endoscopic findings), disease location, use of biologics, medication adherence, treatment duration, surgical indications (e.g., perforation, stricture, or obstruction), surgical types, and genetic factors, may influence the risk of bowel resection and therefore require careful consideration in the interpretation of our findings. For instance, patients undergoing aggressive therapy may experience delayed surgical intervention, while those presenting with acute complications could have distinct proteomic patterns that are not represented in the current dataset. Such missing clinical variables may lead to residual confounding in the estimated risk associations. Third, although the UK Biobank prospectively collects data from participants, it does not specifically target a predefined patient population prior to study initiation. Consequently, certain biases characteristic of retrospective studies remain present. For instance, while over 9000 individuals with IBD were identified in the database, fewer than 1000 had available serum samples for analysis – a discrepancy reflecting inherent collection bias that was not part of the original study design. Additionally, the temporal relationship between sample collection and clinical events was retrospectively assessed. Nevertheless, it is important to note that this study utilizes the UK Biobank resource, which differs fundamentally from conventional single-center retrospective studies in terms of scale, data quality control, and population representativeness. Moreover, although the UKB cohort demonstrates broad population representativeness, the enrollment age restriction (40–69 years) may limit the generalizability of our findings to IBD patients outside this age range, particularly younger individuals and elderly populations. As this study relied on a single database without external validation, confidence in its generalizability to other IBD populations may be limited. The findings should be interpreted with caution until validated. External confirmation in other large-scale prospective cohorts, such as the IBD Plexus and the GEM Project, is needed. Last, translational studies are needed to validate the diagnostic utility of the identified biomarkers (e.g., IL15, PAPPA, and PIK3AP1) and to investigate their mechanistic roles in inflammation and surgical risk in IBD.

## Conclusion

This study demonstrates the potential of Olink proteomics to enhance risk stratification for bowel resection in IBD. By enabling earlier identification of high-risk patients and informing tailored therapeutic strategies, proteomics represents a significant advancement over conventional clinical markers.

## Data Availability

The data used in this study are available from the UK Biobank under approved access. Researchers may apply for access through the UK Biobank portal (https://www.ukbiobank.ac.uk/use-our-data/apply-for-access/).

## Research registration unique identifying number (UIN)

Not applicable. This study is a secondary analysis of existing data from the UK Biobank, and therefore no separate research registration was required.
